# Influence of canopy openness, ungulate exclosure, and low‐intensity fire for improved oak regeneration in temperate Europe

**DOI:** 10.1002/ece3.6092

**Published:** 2020-02-15

**Authors:** Linda K. Petersson, Daniel C. Dey, Annika M. Felton, Emile S. Gardiner, Magnus Löf

**Affiliations:** ^1^ Southern Swedish Forest Research Centre Swedish University of Agricultural Sciences Alnarp Sweden; ^2^ Northern Research Station USDA Forest Service Columbia MO USA; ^3^ Center for Bottomland Hardwoods Research USDA Forest Service Stoneville MS USA

**Keywords:** browsing, burn, disturbance, fire–oak hypothesis, light, *Quercus robur/petraea*, temperate

## Abstract

Failed oak regeneration is widely reported in temperate forests and has been linked in part to changed disturbance regimes and land‐use. We investigated if the North American fire–oak hypothesis could be applicable to temperate European oaks (*Quercus robur*, *Quercus petraea*) using a replicated field experiment with contrasting canopy openness, protection against ungulate browsing (fencing/no fencing), and low‐intensity surface fire (burn/no burn). Survival, relative height growth (RGR_H_), browsing damage on naturally regenerated oaks (≤300 cm tall), and changes in competing woody vegetation were monitored over three years. Greater light availability in canopy gaps increased oak RGR_H_ (*p* = .034) and tended to increase survival (*p* = .092). There was also a trend that protection from browsing positively affected RGR_H_ (*p* = .058) and survival (*p* = .059). Burning reduced survival (*p* < .001), nonetheless, survival rates were relatively high across treatment combinations at the end of the experiment (54%–92%). Most oaks receiving fire were top‐killed and survived by producing new sprouts; therefore, RGR_H_ in burned plots became strongly negative the first year. Thereafter, RGR_H_ was greater in burned plots (*p* = .002). Burning altered the patterns of ungulate browsing frequency on oaks. Overall, browsing frequency was greater during winter; however, in recently burned plots summer browsing was prominent. Burning did not change relative density of oaks, but it had a clear effect on competing woody vegetation as it reduced the number of individuals (*p* < .001) and their heights (*p* < .001). Our results suggest that young, temperate European oaks may respond similarly to fire as their North American congeners. However, disturbance from a single low‐intensity fire may not be sufficient to ensure a persistent competitive advantage—multiple fires and canopy thinning to increase light availability may be needed. Further research investigating long‐term fire effects on oaks of various ages, species‐specific response of competitors and implications for biodiversity conservation is needed.

## Graphical abstract

We investigated if the North American fire–oak hypothesis could be applicable to temperate European oaks. Although a low‐intensity fire reduced oak seedling survival, survival rates were relatively high across treatment combinations (54%–92%) and relative height growth was greater in burned plots after 2 years. Burning did not change relative density of oaks, but it had a clear effect on competing woody vegetation as it reduced the number of individuals and their heights, especially for conifers.

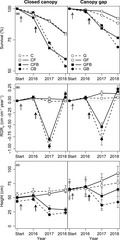

## 1 INTRODUCTION

Present forest composition is the result of historical disturbance regimes, anthropogenic land‐use, climate, and local site conditions. For centuries, natural disturbance and traditional land‐use practices maintained relatively open canopies in many northern temperate forests (Abrams, 1992; Kirby & Watkins, 2015; Vera, 2000). During the 20th century, however, secondary succession following abandonment of past land‐use practices or introduction of modern high‐production forestry changed forest composition and structure, often creating dense forests (Kirby & Watkins, 2015; Nowacki & Abrams, 2008).

Oaks (*Quercus* L.) are typical components of northern temperate ecosystems in which they provide multiple ecosystem services (Johnson, Shifley, Rogers, Dey, & Kabrick, 2018; Löf et al., 2016; Mölder, Meyer, & Nagel, 2019). Due to their disproportionate importance for biodiversity they are foundational species (Dayton, 1972; Ellison et al., 2005), creating crucial habitats for saproxylic invertebrates (Jonsell, Weslien, & Ehnström, 1998), lichens, fungi (Ranius, Eliasson, & Johansson, 2008), and birds (Rodewald & Abrams, 2002). In many areas, however, a combination of changing climate and human activities has favored other woody species over oaks, and thereby, lessened oak abundance relative to previous centuries (Lindbladh & Foster, 2010; McEwan, Dyer, & Pederson, 2011). Furthermore, oak forests face natural regeneration challenges throughout much of their range, which has been linked in part to intensification of forest‐use, increasing ungulate populations, efficient fire suppression, and a lack of sustained management in oak habitats of cultural origin (Bergmeier, Petermann, & Schröder, 2010; Crow, 1988; Dey et al., 2019; Petersson, Milberg, et al., 2019; Shaw, 1968; Watt, 1919).

Though their large acorns allow for seedling establishment in relatively dark understory conditions, oaks are often considered light demanding as they require ample irradiance for survival and growth once energy reserves of the cotyledons are exhausted (Annighöfer, Beckschäfer, Vor, & Ammer, 2015; Johnson et al., 2018). In addition to sufficient light, successful oak regeneration hinges on a variety of other factors that often influence each other, including competing vegetation, ungulate browsing, and site conditions (Annighöfer et al., 2015; Harmer, 2001; Jensen & Löf, 2017; Lorimer, Chapman, & Lambert, 1994). Though knowledge is lacking regarding the combined influence of different disturbance types on suitable environmental conditions, traditional land‐use systems that included grazing regimes, utilization of low‐intensity fire, and cutting of shade‐tolerant trees, likely promoted oak regeneration (Bobiec et al., 2019; Bobiec, Reif, & Öllerer, 2018; Vera, 2000).

In eastern North America, failed oak regeneration has been linked in part to changed disturbance regimes such as fire suppression during the last century (Abrams, 1992; Brose, Schuler, Lear, & Berst, 2001; Dey et al., 2019). This led to development of the fire–oak hypothesis, which states that fire has been an integral part of temperate oak ecosystems and that oaks have ecological traits (e.g., high sprouting capacity and thick bark) that make them better adapted to survive and benefit from periodic fires compared to other hardwood species (Abrams, 1992; Arthur, Alexander, Dey, Schweitzer, & Loftis, 2012; Brose et al., 2001; Nowacki & Abrams, 2008). Thus, prescribed burns along with stand thinning to increase light availability are used extensively in the United States to restore and regenerate oak ecosystems (Brose, Dey, & Waldrop, 2014; Dey & Hartman, 2005). Depending on stage of life and fire regimes, oaks can be classified as fire resisters and endurers (Rowe, 1983). Because of the complexities of fire regime effects on survival and mode of regeneration that complicate classification by Rowe (1983), we, hereafter, refer to oaks as being fire adapted.

In Europe, modern‐day forest fires have mainly been associated with the boreal and Mediterranean zones, and studies of fire effects on oak regeneration have almost entirely been conducted in Mediterranean forests with varying results (e.g., Catry, Moreira, Cardillo, & Pausas, 2012; Monteiro‐Henriques & Fernandes, 2018). However, reconstructions of past forest conditions suggest that fire may have had an important role in the dynamics of certain temperate European forest types as well (Bradshaw & Lindbladh, 2005; Niklasson, Lindbladh, & Björkman, 2002; Niklasson et al., 2010; Tinner, Conedera, Ammann, & Lotter, 2005). In western Ukraine, woodland fires may effectively prevent understory development of hazel (*Corylus avellana* L.), hornbeam (*Carpinus betulus* L.), and beech (*Fagus sylvatica* L.), thereby favoring pedunculate oak (*Quercus robur* L.) reproduction (Bobiec et al., 2019; Ziobro et al., 2016). Moreover, Adámek, Hadincová, and Wild (2016) found that survival rates of sessile oak [*Quercus petraea* (Matt.) Liebl.] trees following fire in central Europe was notably high and almost unaffected by fire intensity. A recent greenhouse experiment revealed that seedlings of *Q. robur* responded similarly to the fire adapted North American white oak (*Q. alba* L.) when they experienced shoot destruction typically sustained during fire (Petersson, Löf, Jensen, Chastain, & Gardiner, 2019). Together, these findings suggest that European temperate oak species (*Q. robur* and *Q. petraea)* may respond to fire in a similar fashion as their North American congeners.

Against this background, we investigated how the combined effects of different disturbances influence natural oak regeneration, and if the North American fire–oak hypothesis could be applicable during the regeneration phase of temperate European oak forests. A replicated field experiment using naturally regenerated oaks and a randomized block design with split–split plots including contrasting canopy openness, protection against browsing ungulates, and low‐intensity surface fire was established in southern Sweden. We hypothesized that: (a) greater light availability would increase tolerance of natural oak reproduction to disturbance; (b) protection against browsing would improve oak growth and survival; and, (c) oaks would survive a low‐intensity surface fire by producing new sprouts. Further, we evaluated the response of competing woody vegetation following the low‐intensity fire.

## 2 MATERIAL AND METHODS

### 2.1 Study sites and species

**Figure 1:**
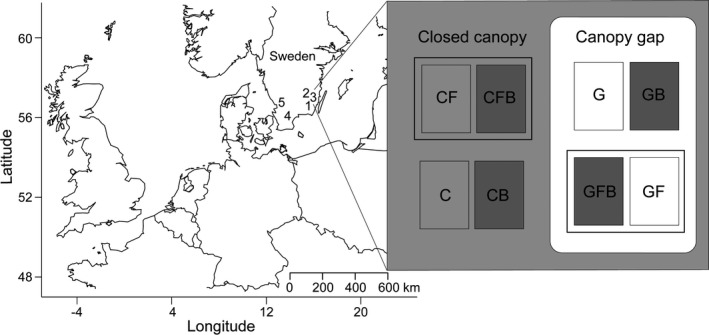
Experimental design including location of the five sites (blocks) in southern Sweden: (1) Abbetorp; (2) Barnebo; (3) Hornsö; (4) Sösdala; and, (5) Sperlingsholm. The enlarged figure shows one block with eight split–split plots, that is, treatment combinations: closed canopy (C), closed canopy and fence (CF), closed canopy, fence, and burn (CFB), closed canopy and burn (CB), canopy gap (G), canopy gap and fence (GF), canopy gap, fence, and burn (GFB) and canopy gap and burn (GB)

The experiment was conducted in five oak‐dominated forests, 45–95 m above sea level, in southern Sweden (Figure 1; Table 1). Southern Sweden consists of a mosaic landscape of forests, farmlands, and lakes. Due to active forest management favoring conifers, the dominant tree species in the region are Norway spruce (*Picea abies* L. Karst) and Scots pine (*Pinus sylvestris* L.). Two ecologically and morphologically similar oak species are native to the region, *Q. robur* and *Q. petraea*. The species naturally hybridize and are sometimes taxonomically treated as two subspecies under *Q. robur* (Roloff, Bärtels, & Schulz, 2008). In this study, they were treated as one species. At the start of the experiment, all study sites had naturally regenerated oak seedlings and saplings in the understory (Table S1).

**Table 1:** Characteristics of the five study sites

Sites	Species composition	Height^a^ (m)	Basal area^a^ (m^2^/ha)		PACL (%)		Moose density^b^ (animals/km^2^)	Deer density^b^ (animals/km^2^)
			Closed canopy	Canopy gap	Closed canopy	Canopy gap		
1. Abbetorp	*Quercus robur/petraea*,* Corylus avellana*,* Tilia cordata*	20	22	5	22 ± 1	35 ± 2	0.5–1.2	1.0–5.3
2. Barnebo	*Q. robur/petraea*,* Picea abies*,* Pinus sylvestris*	21	27	16	23 ± 1	39 ± 1	0.4–1.5	3.2–4.2
3. Hornsö	*Q. robur/petraea*,* Acer platanoides*,* P. abies*	17	29	21	20 ± 1	38 ± 2	0.5–0.7	5.2–8.3
4. Sösdala	*Q. robur/petraea*,* Sorbus aucuparia*,* Betula pendula*	19	28	6	19 ± 1	39 ± 2	0.0–0.9	5.5–15.0
5. Sperlingsholm	*Q. robur/petraea*, *P. sylvestris*,* P. abies*	21	24	9	19 ± 1	45 ± 1	0.0–0.1	2.5–4.5

Note

The three most frequent tree species (listed with respect to their abundance), mean height and basal area for all trees >10 cm dbh in August 2018; percentage of the above canopy light (PACL) in June averaged across the three study years; densities of moose and three deer species during the winter of 2016/2017 and 2017/2018.

a Measurements conducted in a circular plot with a 10 m radius.

b Densities are estimated based on pellet counts, see Appendix S1.

Four browsing ungulate species of the family Cervidae occur in southern Sweden: moose (*Alces alces* L.), roe deer (*Capreolus capreolus* L.), red deer (*Cervus elaphus* L.), and fallow deer (*Dama dama* L.). Moose and roe deer are common and likely responsible for most browsing damage in this study. Also, two species of hare (*Lepus timidus* L. and *L. europaeus* Pallas) may cause browsing damage.

### 2.2 Experimental design and treatments

We used a randomized block design with split–split plots and five blocks, that is, sites (Figure 1). The main treatment was canopy manipulation to create different light levels (closed canopy or canopy gap), with protection against browsing (no fence or fence) nested within the canopy treatment, and a low‐intensity surface fire (no burn or burn) nested within the fence treatment. This created eight treatment combinations: closed canopy (C), closed canopy and fence (CF), closed canopy, fence, and burn (CFB), closed canopy and burn (CB), canopy gap (G), canopy gap and fence (GF), canopy gap, fence, and burn (GFB), and canopy gap and burn (GB). The size of a split–split treatment plot was 25 m^2^, with a distance of 25–188 m and 7–42 m between canopy‐ and fence‐treatments, respectively.

In April 2016, cutting to create canopy gaps of about 400 m^2^ was performed at each site, and a 2‐m steel wire fence was erected around two adjacent, randomly selected plots in each canopy treatment. Cutting was conducted with a chainsaw to remove all large canopy trees in the canopy gaps, but saplings, seedlings, and shrubs were not cut. The fence excluded all ungulates and hares, but provided free access to rodents (mesh size 5 × 5 cm from 0–0.8 m, 16 × 20 cm from 0.8–2.0 m). Two plots in each canopy treatment were thereafter randomly selected for the burn treatment, one inside and one outside the fence. All oak recruits, defined as seedlings, saplings, or sprouts, within browsing height ≤ 300 cm tall (Nichols, Cromsigt, & Spong, 2015) in treatment plots were marked with a uniquely numbered aluminum tag. In plots with > 100 oak recruits, a random subset of at least 50 were chosen for measurements. In total, 2,357 oak recruits were measured through the duration of the experiment (Table S1).

Burning was performed between 29 September and 7 October 2016, following an unusually warm and dry late summer and autumn (Table S2). Delaying the burn to these dates allowed understory vegetation time to acclimate to canopy manipulation and fencing for one growing season prior to burning. To ensure similar burn treatment across sites and plots, we used a propane fired blowtorch (121960L, Kemper) to simulate a low‐intensity surface fire. Plots were systematically burned from one side to the other so that herbaceous vegetation and the top of the fine litter layer was burned (Table S3), but larger pieces of litter and shrubs were charred. Flame heights were visually estimated to be <0.4 m at all sites. It took about one hour to treat one plot and all four plots per site were treated during the same day. Environmental conditions and litter depth were recorded before and after each burn (Table S3).

### 2.3 Measurements

Height (±1 cm) and basal diameter (±1 mm) of all oak recruits were recorded at the start of the experiment in April 2016 (Table S1), and in late August 2016, 2017, and 2018. There were no significant difference in height nor basal diameter between treatments at the start of the experiment (Table S4). In each measurement period, we noted if an oak was alive and the number of stems per individual. For oaks with multiple stems, height and basal diameter were recorded for the tallest stem.

Browsing damage, that is, herbivore removal of twigs, shoots, or buds, was recorded in each year in both April and August 2016–2018. In April 2017, that is, the spring following burning, browsing damage could not be recorded in burn plots as most oaks were top‐killed by the fire and, therefore, had no shoots available for browsers. When possible, the herbivore (hare or ungulate) responsible for damage was determined following Kullberg and Bergström (2001). We defined browsing frequency as the proportion of browsed oaks per treatment plot. Fences remained intact throughout the experiment, and browsing damage did not occur inside fences. Densities of moose and the three deer species were estimated using pellet counts (Table 1, Appendix S1) (Eberhardt & Van Etten, 1956; Månsson, Andrén, & Sand, 2011).

In August of each year in 2016–2018, competing woody vegetation ≤300 cm tall was recorded in four circular 2 m^2^ subplots randomly placed in each treatment plot. Height (±1 cm) and number of individuals per species were recorded, as well as the number of oaks per subplot. These measurements included seedlings that established during the course of the experiment. As oaks dominated the understory at most sites, woody vegetation was combined into species groups: conifers, broadleaves excluding oaks, and oaks.

In late June for each year of 2016–2018, light availability at 160 cm above ground level was estimated using hemispherical imagery (Table 1). One photograph was taken in the middle of each treatment plot on an overcast day (Nikon Coolpix 8800VR, fisheye lens LC‐ER2). The camera lens was oriented perpendicular to the forest floor, and magnetic north was referenced in the image. Images were thresholded in the blue color plane, and percentage of above canopy light reaching the camera was calculated using Gap Light Analyzer software (Frazer, Canham, & Lertzman, 1999).

### 2.4 Calculations and statistical analysis

To account for height variation in oak recruits at the start of the experiment and any size‐related differences in growth rates, we calculated relative height growth rate per year (RGR_H_) for each oak following Hunt (1982):

$${\text{RGR}}_{H}=\frac{\ln\left(H_{2}\right)-\ln\left(H_{1}\right)}{t_{2}-t_{1}}$$

where *H*_2_ and *H*_1_ were oak height the year of interest and the previous year, respectively, *t*_2_ and *t*_1_ were the year of interest and the previous year (in this work *t*_2−_ *t*_1_ was always one year). We then calculated average RGR_H_ per treatment plot. Data from the last year of the experiment, that is, August 2018, were used in the statistical analyses. We analyzed RGR_H_ using a linear mixed‐effects model with the package “lme4” (Bates, Maechler, Bolker, & Walker, 2015), with the following factors (treatments) and their interactions as fixed effects: canopy openness, fence, and burn (all binary). To account for the hierarchical design of the experiment, we included nested (site/light/fence) random effects in the model. Residual and random effect distributions were examined graphically. A possible outlier was identified, however, when excluded it did not affect model results.

Survival was analyzed using a generalized linear mixed‐effects model with binomial error distribution, using a similar model structure (i.e., fixed and random effects) as described for the RGR_H_ model. To investigate whether initial plant size affected survival after burning, an additional survival model using the fixed effects canopy openness, fence (both binary), and initial basal diameter (continuous), and only including the burn treatment, was analyzed. We tested for overdispersion and residual distribution using “testDispersion” and “testUniformity” functions from the DHARMa package (Hartig, 2019).

We analyzed the change of competing woody vegetation, that is, number of plants, using a generalized linear mixed‐effects model with Poisson error distribution, using the following factors as fixed effects: canopy openness, fence, burn (all binary), and species group (three levels). We included nested (site/light/fence/burn) random effects and the initial number of plants per species group as an offset variable. A postanalysis of deviance (Wald *χ* ^2^ Type III) revealed no significance of light or fence as predictor variables, hence we simplified the model using only burn and species group as predictors. This was supported by Akaike's Information Criterion (AIC). Overdispersion and residual distribution were tested as described above. Relative density of oaks, that is, number of oaks divided by total number of plants, and height of competing vegetation (log‐transformed) were analyzed using linear mixed‐effects models as described for the RGR_H_ model.

As ungulate browsing pressure may vary over time, due to, for example, fluctuating ungulate population densities and season, data from the three measurement occasions after burning (August 2017, April 2018, August 2018) were modeled separately to analyze browsing frequency on oak. We used generalized linear mixed‐effects models with binomial error distribution, as described for the survival model, only including the treatment without fences and only ungulate browsing (not hare).

All analyses were performed using R version 3.5.0 (R Core Team, 2017). For all models, AIC was used to identify whether interactions between fixed effects were justified. Significance was determined at an alpha level of 0.05.

## 3 RESULTS

For oak survival and growth, as well as the development of competing woody vegetation, the statistical analyses presented here are based on data from the last year of the experiment (August 2018).

### 3.1 Survival

**Figure 2:**
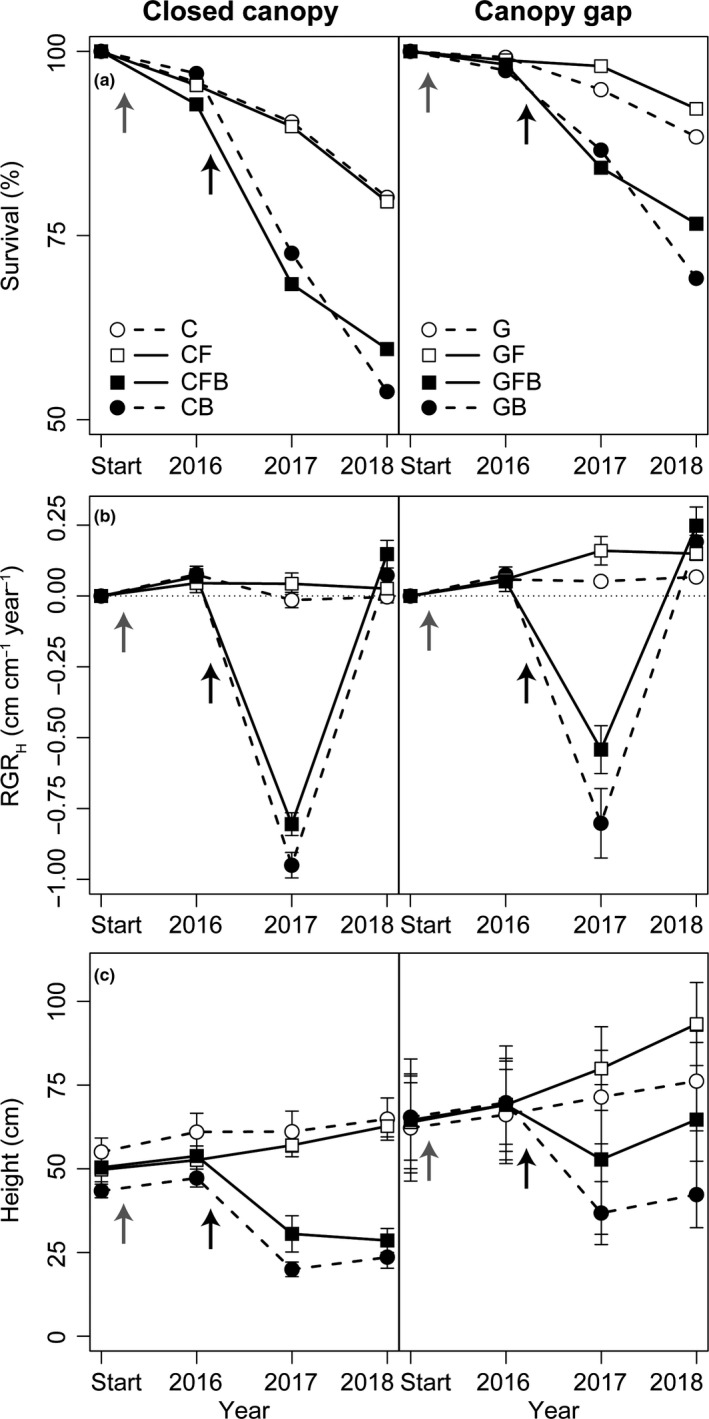
Development of naturally regenerated oaks as (a) survival, (b) relative height growth (RGR_H_), and (c) absolute height under eight treatment combinations [closed canopy (C), closed canopy and fence (CF), closed canopy, fence, and burn (CFB), closed canopy and burn (CB), canopy gap (G), canopy gap and fence (GF), canopy gap, fence, and burn (GFB), and canopy gap and burn (GB)]. Time of canopy gap creation and fencing is indicated with a gray arrow, and time of burn with a black arrow

Survival of naturally regenerated oak recruits declined in all treatment combinations over the course of the experiment, ranging between 54% (CB treatment) and 92% (GF treatment) at the end of the experiment (Figure 2a). No interactions between effects were identified through AIC testing. Though not significant at *α* < 0.05, canopy openness (*χ* ^2^ = 2.83, *df* = 1, *p* = .092) and protection against browsing (*χ* ^2^ = 3.57, *df* = 1, *p* = .059) tended to increase survival. Burning, however, had a significant negative effect on survival (*χ* ^2^ = 141.43, *df* = 1, *p* < .001). The majority of oak recruits affected by fire were top‐killed and survived by producing new sprouts the following year; 88%–96% of surviving oaks affected by burning were resprouts at the end of the experiment. Furthermore, analysis of survival among oaks receiving the burn treatment revealed greater survival for recruits with larger initial stem basal diameter (*χ* ^2^ = 40.02, *df* = 1, *p* < .001), and for recruits growing in a canopy gap (*χ* ^2^ = 4.56, *df* = 1, *p* = .033), while protection against browsing had no significant effect (*χ* ^2^ = 2.51, *df* = 1, *p* = .114).

### 3.2 Growth

As most oaks recruits that survived burning sprouted after top‐kill, their relatively small shoots resulted in a strongly negative RGR_H_ one year after burning (Figure 2b). On average, oaks that sprouted produced 2.6 ± 0.1 shoots. RGR_H_ was greatest under canopy gaps (*F*_1,4_ = 10.10, *p* = .034), and, though not statistically significant, there was a trend that protection from browsing had a positive effect (*F*_1,9_ = 4.71, *p* = .058). Furthermore, RGR_H_ was greater for oaks receiving the burn treatment than those that did not receive burning (*F*_1,19_ = 13.65, *p* = .002). AIC testing supported no interactions between treatments. Despite their greater RGR_H_, height of oak recruits subjected to burning remained lower through the experiment than those that were not burned (Figure 2c). By the end of the study, oaks had not grown above browsing height regardless of treatment.

### 3.3 Competing woody vegetation

Competing woody vegetation (number) was reduced by the burn treatment (*χ* ^2^ = 21.98, *df* = 1, *p* < .001). Moreover, differences were observed between species groups (*χ* ^2^ = 32.00, *df* = 2, *p* < .001). Burning reduced conifers from 750 to 125 plants/ha after two years, broadleaves (excluding oaks) from 9,812 to 5,500 plants/ha, and oaks from 51,652 to 38,250 plants/ha (Table S5). However, the relative density of oaks was not affected by treatment factors (Table S6).

Height of competing vegetation (broadleaves and conifers combined) was not affected by canopy openness (*F*_1,4_ = 0.20, *p* = .681; Table 2). There was a trend, though not significant at *α* < 0.05, suggesting protection against browsing increased competitor height (*F*_1,9_ = 4.71, *p* = .058). Concurrently, competitor height was lowest where plots were burned (*F*_1,19_ = 23.36, *p* < .001).

**Table 2:** Height of competing woody vegetation in August 2016 and 2018 (mean ± *SE*, *n* = 5). Heights in 2018 also include individuals that were established after measurements in 2016

Treatment	Height (cm)	
	2016	2018
Closed canopy (C)	95 ± 22	105 ± 26
Closed canopy and fence (CF)	51 ± 12	71 ± 13
Closed canopy, fence, and burn (CFB)	76 ± 17	45 ± 3
Closed canopy and burn (CB)	94 ± 19	37 ± 5
Canopy gap (G)	40 ± 4	55 ± 9
Canopy gap and fence (GF)	73 ± 12	117 ± 10
Canopy gap, fence, and burn (GFB)	71 ± 15	60 ± 7
Canopy gap and burn (GB)	58 ± 19	39 ± 5

### 3.4 Browsing frequency

**Figure 3:**
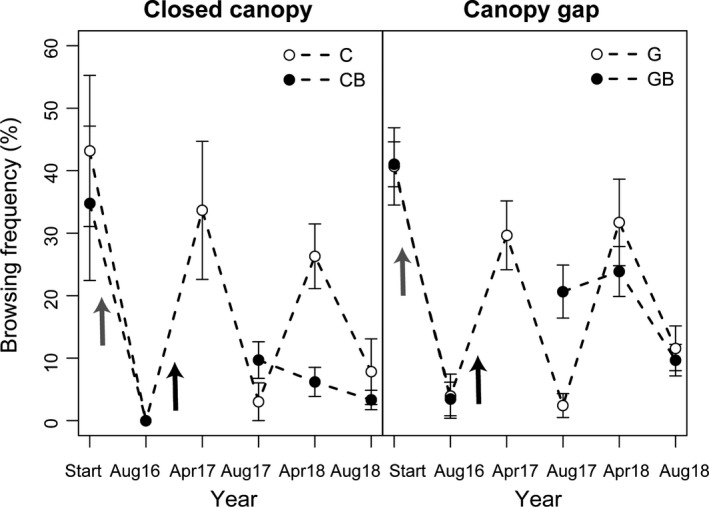
Ungulate browsing frequency (proportion of browsed oaks per plot) on naturally regenerated oaks in four treatment combinations [closed canopy (C), closed canopy and burn (CB), canopy gap (G), and canopy gap and burn (GB)]. Measurements in April correspond to preceding winter browsing, measurements in August correspond to preceding summer browsing. Time of canopy gap creation is indicated with a gray arrow, and time of burn with a black arrow

The majority of browsing damage was caused by ungulates; hares were responsible for < 5% of all observed browsing damage. Overall, ungulate browsing frequency on oak recruits was greater during winter than summer (Figure 3). Burning, which was applied in the autumn of 2016, temporarily influenced the pattern of browsing frequency. In August 2017 (one year after burning), browsing frequency was greatest in the burned plots (Figure 3, Table 3), presumably a response to the new sprouts that emerged in the summer of 2017. Thereafter, browsing frequency was greatest in nonburned plots (Figure 3, Table 3). Except for the winter 2017/2018, browsing frequency was unaffected by canopy openness. In that winter, an interaction between the canopy and burn treatments suggested that browsing frequency was lowest in the CB treatment.

**Table 3:** Analysis of deviance table based on a generalized linear mixed‐effects model (binomial) explaining ungulate browsing frequency on naturally regenerated oak recruits among treatments and their interactions (excluding fence treatment). Browsing frequency measured in August corresponds to preceding summer browsing, measurements in April corresponds to preceding winter browsing

Factor	*χ* ^2^	*df*	*p*
Aug 2017			
Canopy openness	0.97	1	.325
Burn	37.02	1	<.001
Canopy openness: burn	3.19	1	.074
Apr 2018			
Canopy openness	8.07	1	.005
Burn	30.14	1	<.001
Canopy openness: burn	10.32	1	.001
Aug 2018			
Canopy openness	3.67	1	.056
Burn	5.63	1	.018
Canopy openness: burn	3.58	1	.058

Note

Type III Wald *χ* ^2^ tests.

## 4 DISCUSSION

We investigated if the fire–oak hypothesis (Abrams, 1992; Arthur et al., 2012) is applicable to European temperate oaks by establishing a field experiment with contrasting canopy openness, protection against wild ungulate browsers, and a low‐intensity fire. The experimental design allowed us to examine potential combined effects of these disturbance‐related factors. At the end of the experiment, oak survival was high across treatment combinations, and relative height growth rate (RGR_H_) was greatest where burning was applied. Furthermore, the relatively higher light availability within canopy gaps increased RGR_H_ and oak survival where fire was prescribed. Burning also clearly effected competing woody vegetation by reducing the number of individuals, though it did not change relative density of oaks. These results, which are similar to reports from North American studies (e.g., Brose, Dey, Phillips, & Waldrop, 2013; McEwan et al., 2011), could indicate a common ecological mechanism impactful to the dynamics of oak regeneration in temperate forests across the Atlantic.

Though fire reduced survival of naturally regenerated oaks, survival rates were still relatively high across treatments. This high survival was largely dependent on new shoot production following top‐kill. Top‐kill initially decreased RGR_H_, but by the end of the experiment sprouting resulted in a vigorous RGR_H_. One reason many North American oaks are considered fire adapted is the relatively greater sprouting capacity they exhibit over their competitors (Abrams, 1992; Brose et al., 2014). The high sprouting capacity and increased growth rate of *Q. robur/petraea* observed in this field experiment supports the hypothesis that natural regeneration of temperate European oaks could be promoted by low‐intensity fire. However, long‐term observations are needed to determine if the positive fire effects noted through this research will persist over time.

As expected, oak RGR_H_ was greater in canopy gaps than under closed canopies. However, oak survival was not significantly affected by canopy openness, except where burning was applied. *Q. robur* and *Q. petraea* require a minimum of 15%–20% of full light for sustained growth (Löf, Karlsson, Sonesson, Welander, & Collet, 2007; von Lüpke, 1998). This corresponds to the available light beneath closed canopies in this study and can probably explain the high survival rates of the oaks growing there. Nevertheless, the observation that survival after prescribed burning was improved in canopy gaps suggests that oak resilience to disturbance (which is conferred by sprouting) is enhanced by light. North American studies have shown that prescribed burning offers the greatest to oak regeneration when conducted in combination with overstory treatments that increase light availability (e.g., Brose et al., 2013; Hutchinson, Long, Rebbeck, Sutherland, & Yaussy, 2012). Improving light availability the year prior to burning in this experiment likely favored development of belowground carbohydrate reserves by oaks, which supported sprouting and rapid growth following the prescribed fire (Kabeya & Sakai, 2005; Welander & Ottosson, 1998). Furthermore, the observation of survival being greatest for oaks with the largest stem diameters is consistent with previous research (Dey & Hartman, 2005).

Though oaks are preferentially browsed by ungulates (Bergqvist, Wallgren, Jernelid, & Bergström, 2018), they are considered browsing tolerant because they can survive moderate browsing for extended periods (Harmer, 2001). However, browsing can severely limit height growth and high browsing pressure can prevent oak and other palatable tree species from advancing to the overstory (Churski, Bubnicki, Jedrzejewska, Kuijper, & Cromsigt, 2016; Rooney & Waller, 2003). We observed a trend indicating that protection from wild ungulate browsers positively affected oak recruit survival and RGR_H_. Considering the relatively short duration of our experiment, it was not surprising that protection against browsing had a somewhat limited impact. Additionally, there was considerable variation in browsing animal density between study sites and years, so it is possible that this variation masked positive effects of protection against browsers in this study.

According to the fire–oak hypothesis, many oaks have traits that confer a competitive advantage over other tree species following periodic fires (Abrams, 1992; Arthur et al., 2012). Testing the fire–oak hypothesis on temperate European oaks should therefore also include an assessment of fire effects on competing vegetation. In this study, woody vegetation was assessed as species groups since oak seedlings and saplings dominated the understory on most sites. This limits our ability to distinguish species‐specific responses of oak competitors. Furthermore, it was not possible to distinguish sprouts from newly established seedlings. Nevertheless, we observed clear fire effects on competing woody vegetation as prescribed burning reduced the number of individuals, especially the number of conifers (*P. abies* and *P. sylvestris*). This was expected because these conifers lack the ability to sprout when top‐killed (Leonardsson & Götmark, 2015). It is, however, worth noting that there were fewer conifers present on the study sites as compared to broadleaves (Table S5). The height of competing vegetation was also lowered in burned plots, but competing vegetation remained roughly the same height or slightly taller than oak reproduction at the end of the experiment.

Our review of literature from the eastern United States indicated that a single, dormant season prescribed fire is often not sufficient to favor oak regeneration (McEwan et al., 2011). Rather multiple fires have been more successful (Dey & Hartman, 2005; Hutchinson et al., 2012). This corresponds to Ziobro et al. (2016) and Bobiec et al. (2019), who reported that reoccurring grass burning promoted *Q. robur* regeneration by reducing competing shade‐tolerant species in Ukrainian woodlands. We, therefore, suggest that additional experiments involving multiple burning events are needed to determine whether reoccurring fire provides *Q. robur* and *Q. petraea* competitive advantage. The relatively small burn treatments in this study allowed us to experimentally examine potential combined effects of multiple disturbance‐related factors. Nevertheless, natural wildfires or prescribed burns generally impact larger areas, and the varying fire intensity of fire over larger areas can often result in pockets of unburnt vegetation (e.g., Lampainen, Kuuluvainen, Wallenius, Karjalainen, & Vanha‐Majamaa, 2004). Future studies investigating the effects of larger fire events on oak regeneration would, therefore, be of interest. Furthermore, the timing of a fire is known to affect how vegetation responds after fire. North American studies have demonstrated that growing season fires have greater impact than do dormant season fires because vegetation is physiologically active (Brose et al., 2014). Though burning was conducted at the very end of the growing season in this study, it is likely that the timing of the burns we applied impacted plants that were physiologically active.

Fire changed the pattern of ungulate browsing frequency. While the peak in browsing frequency was during the winter, summer browsing was also prominent where plots were recently burned. A similar observation has been reported for the United States (e.g., Andruk, Schwope, & Fowler, 2014; Collins & Carson, 2003). Considering the relatively small treatment plots of this study, it seems unlikely the increased browsing frequency was caused by a decrease in availability of alternative forage. Rather it may have resulted from changed foliar nutrient concentrations and/or foliar defensive chemistry that increased palatability of postfire–oak sprouts (Reich, Abrams, Ellsworth, Kruger, & Tabone, 1990; Rieske, 2002). This could also explain why browsing frequency was lower in the burn treatments compared to the nonburned treatments two years after the fire, as both Reich et al. (1990) and Rieske (2002) found increases in foliar nutrients resulting from prescribed burning diminished over time.

Considering most north temperate ecosystems have experienced dramatic increases in ungulate populations over the last few decades (Milner et al., 2006; Rooney & Waller, 2003) the interaction between fire and browsing frequency, we observed is particularly notable. It suggests that positive effects of prescribed fire on oak regeneration could be diminished in forests subject to high browsing pressure. In other words, oaks may readily survive moderate browsing pressure (Harmer, 2001), but browsing in combination with fire is likely to be more detrimental to survival of oak recruits. Indeed, Nuttle, Royo, Adams, and Carson (2013) found that high browsing pressure reduced the benefits of fire and canopy gap creation on tree diversity in forest understories.

### 4.1 Conclusion and management implications

Our results indicate that it is vital to increase light availability to promote naturally regenerated oak recruit survival and growth, especially when disturbances such as browsing and/or prescribed fire damage oak shoots. In areas of high conservation concern, this could be accomplished by removing canopy trees of less importance for biodiversity than oaks (cf. Leonardsson, Löf, & Götmark, 2015). Protection from ungulate browsing had a limited impact in this experiment; however, considering ungulate preference for oak browse, it is likely their impact will be maintained or even increase over time. This work also demonstrated relatively high survival and invigorated growth of *Q. robur/petraea* sprouts following a low‐intensity surface fire.

Altogether, our results indicate that a low‐intensity surface fire combined with some form of canopy cutting to increase light availability may serve as a first step for promoting natural regeneration of *Q. robur* and *Q. petraea* in temperate European forests. In particular, broadleaved forests of conservation concern in north temperate Europe, such as those examined in this study, often develop a significant regeneration pool of the shade‐tolerant *P. abies* (Götmark, Berglund, & Wiklander, 2005). The reduction of conifer reproduction that we observed through burning supports the application of low‐intensity fire as an efficient management tool in such situations. Fire would likely also be efficient in situations where shade‐tolerant *F. sylvatica* and *C. betulus* constitute the main competitors, which is often the case in central temperate Europe (Bobiec et al., 2019; Ziobro et al., 2016). However, further research is needed in several areas before we can recommend prescribed burns in oak‐dominated forests. Long‐term vitality of oak regeneration and overstory oaks following fire needs to be evaluated, preferably within different light environments and in areas with varying browsing pressure to further elucidate combined effects. Also, an understanding of the possible benefits of multiple fires, as well as evaluation of fire frequency and seasonality, is needed. Furthermore, the species‐specific response of competing woody vegetation following fire needs further investigation to determine whether oaks gain a competitive advantage. Finally, as oak‐dominated forests are of great importance for conservation of biodiversity, the effects of fire on species requiring oak habitats needs thorough examination.

## AUTHOR CONTRIBUTIONS

All authors contributed to conceive the ideas and designed methodology; LP collected and analyzed the data; LP and ML led writing of the manuscript. All authors contributed critically to drafts and gave final approval for publication.

## CONFLICT OF INTETEST

None declared.

## Data Availability

Data are available at Dryad Digital Repository: https://doi.org/10.5061/dryad.cz8w9gj0h.
