# Supplementary material for: Influence of canopy openness, ungulate exclosure, and low‐intensity fire for improved oak regeneration in temperate Europe
Source: Ecol Evol. 2020 Feb 15;10(5):2626–37. doi: 10.1002/ece3.6092 (PMC7069284; doi:10.1002/ece3.6092)
Supplement: Supplementary file 1 [file ECE3-10-2626-s001.docx]

**Appendix**

*Method, pellet counts*

Pellet counts were adapted from Eberhardt and Van Etten (1956), Månsson, Andrén and Sand (2011), and <https://www.slu.se/globalassets/ew/org/centrb/algforvaltning/manualer/adaptiv-algforvaltning-2019/m3_spillningsinventering_2019.pdf> (manual in Swedish). A 1 x 1 km square was placed around each experimental site with 20 circular sample plots every 200 m (if necessary, due to e.g. roads and lakes, plots were moved sideways up to 200 m). The center of sample plots were permanently marked with a 1 m woody stick. Moose sample plots were 100 m^2^ (radius 5.64 m) and deer sample plots were 10 m^2^ (radius 1.78 m). All pellets were cleaned from plots in autumn and surveyed the following spring. Only pellet groups including 20 or more single pellets from moose and 10 or more single pellets from deer with the majority of pellets inside the sample plot were counted. We combined the three deer species (roe deer, red deer, and fallow deer) as their pellets are difficult to distinguish in the field (Spitzer *et al.* 2019). Animal density (km^-2^) for each study site was calculated for moose and the three deer species as:

$$Animal density=\frac{PG*k}{NP*D*T}$$

where PG is the total number of pellet groups, k is an area scaling factor (10 000 for moose, 100 000 for deer), NP is the number of surveyed sample plots, D is the defecation rate (17 for moose, 22 for deer), and T is the number of days between autumn cleaning and spring survey.

Table S1. Initial height, basal diameter (mean ± SE) and number of measured oak recruits (≤300 cm tall) per treatment combination at the start of the experiment in April 2016 (n = 5).

| Treatment | Height (cm) | Basal diameter (mm) | No. oak recruits |
| --- | --- | --- | --- |
| Closed canopy (C) | 55±3 | 8±0.3 | 262 |
| Closed canopy and fence (CF) | 50±2 | 8±0.3 | 302 |
| Closed canopy, fence and burn (CFB) | 51±2 | 8±0.3 | 318 |
| Closed canopy and burn (CB) | 43±2 | 8±0.2 | 273 |
|  |  |  |  |
| Canopy gap (G) | 61±3 | 9±0.4 | 288 |
| Canopy gap and fence (GF) | 68±3 | 9±0.3 | 303 |
| Canopy gap, fence and burn (GFB) | 65±3 | 9±0.3 | 323 |
| Canopy gap and burn (GB) | 62±3 | 9±0.3 | 288 |

Table S2. Average growing season temperature and precipitation (min-max values) during the three study years (n = 5). Data comes from the Swedish Meteorological and Hydrological Institute weather stations, 7-40 km and 7-20 km from each study site for temperature and precipitation, respectively.

| Month | 2016 | | 2017 | | 2018 | |
| --- | --- | --- | --- | --- | --- | --- |
|  | Temp. (C°) | Precipitation (mm) | Temp. (C°) | Precipitation (mm) | Temp. (C°) | Precipitation (mm) |
| May | 12-14 | 9-55 | 11-13 | 21-36 | 15-17 | 8-12 |
| June | 16-17 | 32-84 | 14-15 | 84-120 | 17-18 | 21-30 |
| July | 17-18 | 51-76 | 15-16 | 44-93 | 20-21 | 7-30 |
| August | 15-16 | 52-95 | 15-16 | 63-147 | 17-18 | 108-159 |
| September | 14-15 | 7-19 | 12-13 | 77-127 | 13-14 | 33-64 |

Table S3. Environmental and burn conditions during burn application shown as averages for the four burned plots at each site.

| Site | Burn date | Litter depth  reduction  (cm) | Air temp. (C°) | Relative humidity (%) | Wind speed  (m s^-1^) |
| --- | --- | --- | --- | --- | --- |
| 1. Abbetorp | 7 Oct 2016 | 2.9 (50%) | 12 | 68 | 1 |
| 2. Barnebo | 5 Oct 2016 | 1.9 (35%) | 10 | 74 | 1 |
| 3. Hornsö | 6 Oct 2016 | 2.7 (51%) | 10 | 75 | 2 |
| 4. Sösdala | 29 Sep 2016 | 1.4 (30%) | 18 | 84 | 1 |
| 5. Sperlingsholm | 30 Sep 2016 | 1.2 (32%) | 14 | 73 | 1 |

Table S4. Analysis of variance table based on linear mixed-effects models (as described for RGR_H_ in the method section) explaining height (log-transformed) and basal diameter among treatments at the start of the experiment in April 2016.

| Factor | *F* | df | *P* |
| --- | --- | --- | --- |
| Height |  |  |  |
| Canopy openness | 0.78 | 1,4 | 0.428 |
| Fence | 0.00 | 1,9 | 0.975 |
| Burn | 1.30 | 1,19 | 0.268 |
| Basal diameter |  |  |  |
| Canopy openness | 0.53 | 1,4 | 0.507 |
| Fence | 0.15 | 1,9 | 0.709 |
| Burn | 0.13 | 1,19 | 0.724 |

Table S5. The total number of plants (×10^3^ ha^-1^) per species group (conifers, broadleaves excluding oaks, and oaks) and treatment combination in August 2016 and 2018 (n = 5). Individuals, including the number of oaks, were counted in four separate subplots in each measurement plot and include individuals established during the course of the experiment.

| Treatment | Conifers  (×10^3^ ha^-1^) | | Broadleaves  (×10^3^ ha^-1^) | | Oaks  (×10^3^ ha^-1^) | |
| --- | --- | --- | --- | --- | --- | --- |
|  | 2016 | 2018 | 2016 | 2018 | 2016 | 2018 |
| Control (C) | 0.50 | 1.25 | 7.00 | 7.25 | 52.50 | 48.50 |
| Closed canopy and fence (CF) | 1.00 | 1.00 | 8.25 | 7.50 | 45.50 | 49.50 |
| Closed canopy, fence and burn (CFB) | 0.25 | 0 | 5.75 | 3.75 | 53.25 | 37.00 |
| Closed canopy and burn (CB) | 0.25 | 0 | 5.00 | 3.00 | 67.75 | 44.75 |
|  |  |  |  |  |  |  |
| Canopy gap (G) | 0.50 | 0.50 | 14.50 | 14.25 | 42.75 | 37.50 |
| Canopy gap and fence (GF) | 0.50 | 0.50 | 11.00 | 8.75 | 55.50 | 52.75 |
| Canopy gap, fence and burn (GFB) | 1.00 | 0.25 | 13.50 | 9.50 | 43.00 | 38.50 |
| Canopy gap and burn (GB) | 1.50 | 0.25 | 15.00 | 5.75 | 42.50 | 32.75 |

Table S6. Analysis of variance table based on a generalized linear mixed-effects model (poisson) explaining relative density of oaks, i.e. the number of oaks divided with the total number of woody individuals, among treatments and their interactions in August 2018.

| Factor | *F* | df | *P* |
| --- | --- | --- | --- |
| Canopy openness | 0.10 | 1,4 | 0.771 |
| Fence | 0.18 | 1,8 | 0.685 |
| Burn | 0.19 | 1,16 | 0.669 |
| Canopy openness:fence | 0.49 | 1,8 | 0.506 |
| Canopy openness:fire | 2.21 | 1,16 | 0.157 |
| Fence:fire | 1.15 | 1,16 | 0.300 |
| Canopy openness:fence:fire | 0.87 | 1,16 | 0.364 |
